# A Tale of Two Hyper-diversities: Diversification dynamics of the two largest families of lichenized fungi

**DOI:** 10.1038/srep10028

**Published:** 2015-05-06

**Authors:** Ekaphan Kraichak, Pradeep K. Divakar, Ana Crespo, Steven D. Leavitt, Matthew P. Nelsen, Robert Lücking, H. Thorsten Lumbsch

**Affiliations:** 1 Science and Education, The Field Museum, 1400 S Lake Shore Drive, Chicago, IL 60605 USA; 2 Department of Botany, Faculty of Science, Kasetsart University, Bangkok, 10900 Thailand; 3 Departamento de Biologia Vegetal II, Facultad de Farmacia, Universidad Complutense de Madrid, Madrid 28040, Spain; 4 Department of Geological and Environmental Sciences, Stanford University, Stanford, CA 94305, USA

## Abstract

Renewed interests in macroevolutionary dynamics have led to the proliferation of studies on diversification processes in large taxonomic groups, such as angiosperms, mammals, and birds. However, such a study has yet to be conducted in lichenized fungi – an extremely successful and diverse group of fungi. Analysing the most comprehensive time-calibrated phylogenies with a new analytical method, we illustrated drastically different diversification dynamics between two hyper-diverse families of lichenized fungi, Graphidaceae and Parmeliaceae, which represent more than a fourth of the total species diversity of lichenized fungi. Despite adopting a similar nutrition mode and having a similar number of species, Graphidaceae exhibited a lower speciation rate, while Parmeliaceae showed a sharp increase in speciation rate that corresponded with the aridification during the Oligocene-Miocene transition, suggesting their adaptive radiation into a novel arid habitat.

Recent advances in phylogenetic reconstruction and comparative methods have renewed interest in macroevolutionary dynamics of large taxonomic groups, such as in plants^1^,^2^,^3^, mammals^4^, and birds^5^. These studies have provided additional insights into timing and processes of diversification – a research program that has traditionally been pursued from a mostly paleontological perspective^6^. However, these recent methodological advances enable us to shed light on diversification processes in organisms with little or no fossil record.

Lichens – symbiotic associations of fungi with algae and/or cyanobacteria – represent an extremely successful nutritional mode in the fungi, allowing a heterotrophic fungal partner to expand their ecological range without relying on “external” sources of energy. Nearly one-fifth of all fungi are lichen-forming^7^ , and they are estimated to cover roughly 10% of terrestrial ecosystems^8^. The overall success of lichenized state is hypothesised to play a critical role in early terrestrial ecosystems^9^ and, in many cases, was shown to correlate with paleoclimatic events, such as global cooling and drying^10^,^11^.

This successful symbiosis has led to a wide range of growth forms and habitat preferences. Lichens can be found as a small, inconspicuous crusts adhering to leaf surfaces in the tropics, all the way to a large, sturdy turf in Antarctica. A number of studies that investigated the evolution of nutritional modes have found that the lichenized state has repeatedly and independently gained and lost throughout the Ascomycota, a group to which most lichenized fungi belong^12^,^13^,^14^,^15^,^16^. Every loss of the lichenized form appears to almost always follow by a regain, as well as an increase in diversification rates^17^. However, beyond this limited number of studies, our understanding of how different lichenization events contribute to diversification remains largely incomplete.

While they all acquire a photosynthetic partner as part of their symbiosis, not all lineages of lichenized fungi are equally diverse. Within Lecanoromycetes, a group that contains more than 14,000 species of mostly lichen-forming fungi, the numbers of species among the clades are clearly uneven. While the two largest families, Graphidaceae and Parmeliaceae, have around 2,500-2,800 species each^18^,^19^. The third largest family, Verrucariaceae, trails behind with an estimate of circa 870 species with a few other lichen families that approach this level of species richness^7^. If a partnership with photosynthetic organisms is a successful nutritional mode for fungi, why are not all lichenized fungi more or less equally diverse? Do the two largest families share some of the same underlying mechanisms to achieve such a high level of diversity? In this study, we investigated diversification dynamics of the two largest families of lichenized fungi – Graphidaceae and Parmeliaceae – by examining the rates and timing of diversification to ascertain whether similar mechanisms are operating in these two hugely successful lineages of lichenized fungi. We employed the newly proposed Bayesian Analysis of Macroevolutionary Mixture (BAMM)^20^ to detect heterogeneity in speciation rates across the time-calibrated tree, while accounting for non-random incomplete sampling. For each of these two hyperdiverse families, we asked the following questions (1) Is there acceleration in speciation rates within a family? If so, which groups contribute to accelerated rates? (2) When and how often did a rate shift occur?(3) Are the changes in speciation associated with global climate? and (4) with the results from the two families, do they share similar patterns in diversification processes? Despite their unique nature of hyperdiversity among lichenized fungi, we hypothesised that the two largest families of lichenized fungi would have very different diversification patterns due to their differences in growth forms, lineage ages, and geographic distributions. More specifically, we expected to see a more dynamic diversification pattern in Parmeliaceae, because of its higher diversity of growth froms, younger lineage age, and wider geographical distribution than those of Graphidaceae.

## Results

### Lineage-specific diversification rate

**Figure 1:**
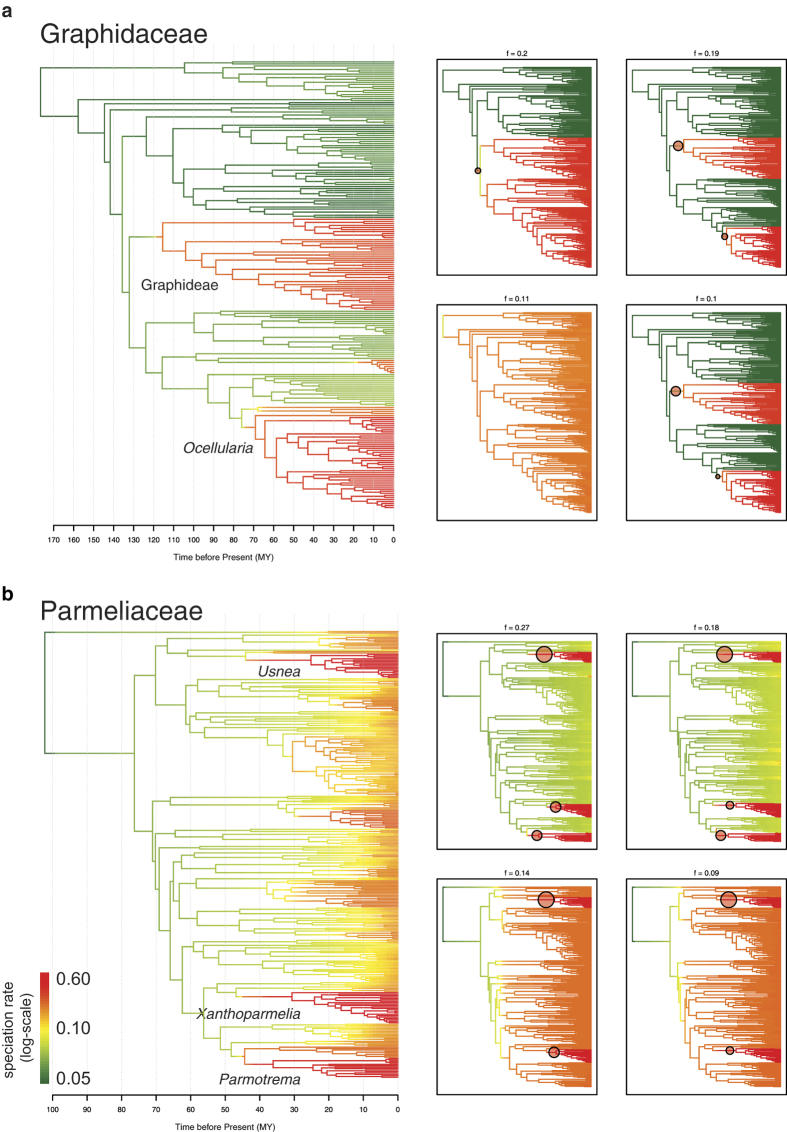
Phylorate plots of lichenized fungal families Graphidaceae (**a**) and Parmeliaceae (**b**). The colours indicate relative speciation rate on each branch on the maximum clade credibility tree. Four panels on the right depict four most common rate shift configurations from the 95% credible shift set with the locations of rate shift indicated by red circles.

Under a relaxed molecular clock model, we estimated the ages of the families Graphidaceae and Parmeliaceae at around 176 and 102 million years ago (MYA), respectively. High levels of heterogeneity in speciation rates were detected across the maximum clade credibility (MCC) trees estimated for each of the families (Fig. 1).

In Graphidaceae, speciation rates ranged between 0.06 and 0.1 lineage/million year (Fig. 1a). Two hyper-diverse clades of the tribes Graphideae and *Ocellularia* s.str. exhibited higher speciation rates than the remainder of the family. Three out of the four most frequent distinct configurations of rate shifts also supported rate shifts either at the common ancestor of Graphideae and *Ocellularia* s.str., or at the stem of each of these clades separately (combined frequency of 0.49). The posterior distribution of the number of shifts suggested two shifts to be the most common configuration (frequency = 0.39).

In Parmeliaceae, the range of speciation rates was between 0.05 and 0.6 lineage/million year (Fig. 1b). The rates were elevated in a number of clades across the family, especially for the genera *Usnea*, *Xanthoparmelia*, and *Parmotrema*. The four most frequent distinct configurations of rate shifts consistently supported at least two rate shifts for the ancestors of the *Usnea* and *Xanthoparmelia* clades (combined frequency of 0.68). The two most frequent configurations also revealed another shift around the crown of the *Parmotrema* clade (combined frequency of 0.45). Three shifts are the most frequent number of shifts found in the posterior distribution (frequency = 0.42).

### Diversification regimes

**Figure 2:**
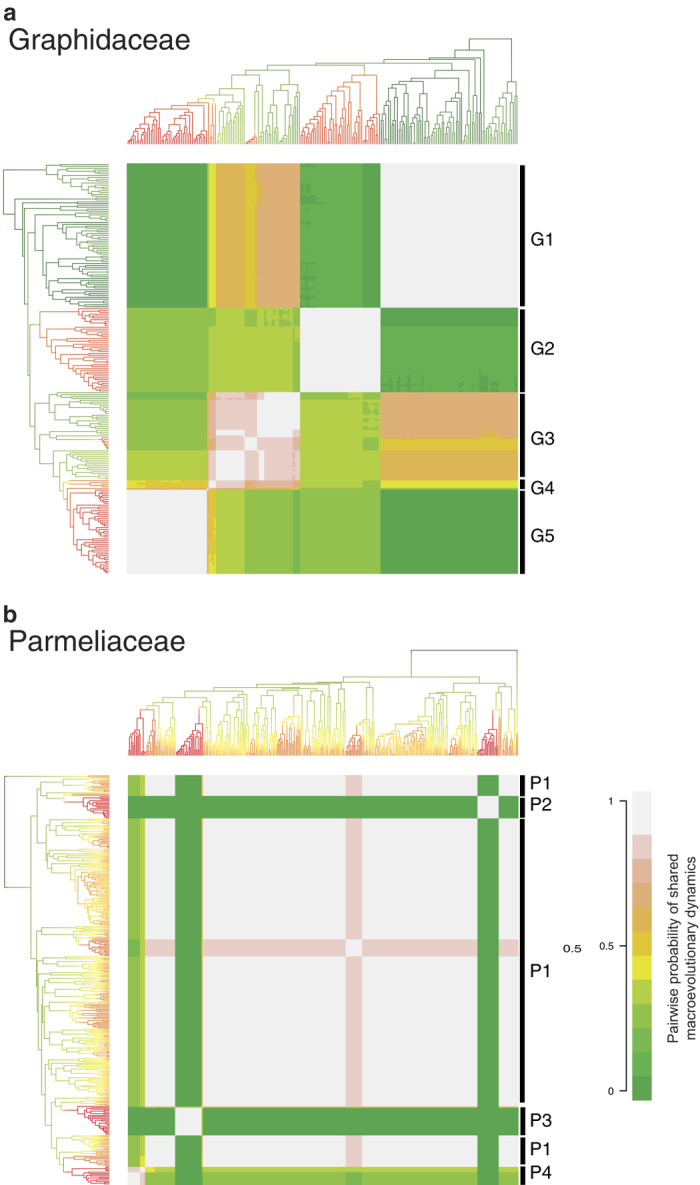
Cohort analyses of lichenized fungal families Graphidaceae (**a**) and Parmeliaceae (**b**). The colours indicate a relative pairwise similarity between speciation rates of two tips across the tree (1 = most similar, 0 = most dissimilar). The letter codes on the right denote different “diversification regimes” in the family.

By computing similarities of rates among the tips of the tree, a cohort analysis revealed a high level of heterogeneity in diversification regimes in Graphidaceae. Upon visual inspection, we categorised the diversification regimes into five groups (denoted on the right side of Fig. 2a). The first regime (G1) was found among the basal lineages of the family, including the members of subfamilies Fissurinoideae, Redonographoideae, and tribe Thelotremateae. The second regime (G2) was limited to the taxa in tribe Graphideae. The third regime (G3) included taxa from three distinct clades from tribes Wirthiotremateae, Diploschisteae, and the *Myriotrema* clade, and appeared to be more similar to G1 than the regimes of closely related lineages (G4, G5). The *Rhabdodiscus* clade constituted the fourth regime (G4), which was at most intermediate in their similarities to other regimes. Lastly, the fifth regime (G5) was unique to the taxa in the *Ocellularia* s.str. clade.

In comparison to Graphidaceae, Parmeliaceae revealed fewer, more homogenous diversification regimes (Fig. 2). The first regime (P1) was the most prevalent in the family with a slight deviation in the *Hypogymnia* clade (shaded pink). The rest of the regimes were unique to the taxa of the hyper-diverse *Usnea* (P2), *Xanthoparmelia* (P3), and *Parmotroma* (P4) clades.

### Correlations of speciation rates and global climate

**Figure 3:**
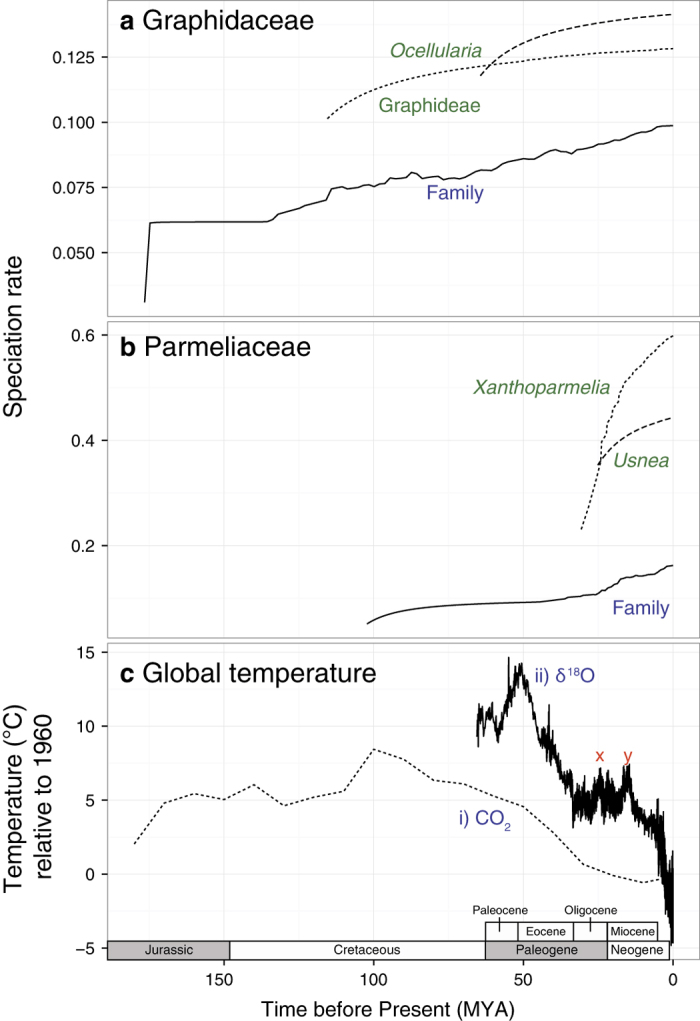
Speciation rate through time plots of lichenized fungal families Graphidaceae (**a**) and Parmeliaceae (**b**) along with the estimates of global temperature anomalies from the past 180 million years ago (**c**) from two proxies: CO_2_ (i) and δ^18^O (ii). The rates of the two lineages with the highest speciation rates from each family were also plotted on the same scale.

For Graphidaceae, the speciation rate increased sharply at the base of the family and then stabilized until around 125 MYA, when the rate increased gradually up to the present time (Fig. 3a). The speciation rates of the two hyperdiverse clades, Graphideae and *Ocellularia* s.str., were markedly higher than the family and appeared to align with two sharp increases in diversification rates at 110 and 65 MYA. No clear correlation with the global temperature was observed. In Parmeliaceae, the rate elevated gradually from the origin of the family and with a sharp increase around 20-25 MYA (between late Oligocene warming and mid-Miocene climatic optimum; Fig. 3c), where the two most diverse clades (*Usnea* and *Xanthoparmelia*) also emerged and started to diversify.

## Discussion

Despite having a similar number of species, the two most diverse families of lichenized fungi showed different diversification patterns. In the older lineage, Graphidaceae, the speciation rate increased rapidly early on and then gradually over the past 150 million years. The younger Parmeliaceae, in contrast, has a higher rate of speciation since the emergence of the family at 102 MYA with a noted increase around 20-25 MYA, which coincided with late Oligocene warming and the rise of the hyperdiverse genera *Usnea* and *Xanthoparmelia*. Therefore, the hyperdiversity of Graphidaceae appears to be the results of long evolutionary history, whereas changes in the global climate seems to have contributed significantly to the hyperdiversity in Parmeliaceae.

A lower average rate of speciation in Graphidaceae is not entirely surprising. A recent study also showed that microlichens – which all of the Graphidaceae species are – tend to have a lower diversification rate than macrolichens, such as Parmeliaceae^17^. Even though the level of diversity in Graphidaceae exceeds that of all other macrolichen lineages except for Parmeliaceae, the age of the family is likely much older than many macrolichen lineages. In our specific case, the origin of Graphidaceae predates that of Parmeliaceae by almost 70 million years, but now each has roughly the same number of species. Several reasons can contribute to the lower rate of speciation in microlichens. First, the generation time appears to be longer in microlichens, as they tend to grow at a much slower rate than macrolichens^21^. Consequently, fast-growing macrolichens can outcompete microlichens by occupying substrate surface, driving microlichens to occur in microhabitats where macrolichens cannot normally occur^22^. The other explanation may simply be that a large proportion of species diversity in Graphidaceae has not yet been described. According to a recent estimate, the number of species in Graphidaceae could exceed 4,000 – nearly twice the current number – if the maximum collecting effort could ever be realised^18^. With more described species, the speciation rate might become more comparable to that of Parmeliaceae. However, the species-level diversity in Parmeliaceae is also likely to be underestimated, and continuing taxonomic research for both families will be central to future comparisons.

High heterogeneity in diversification regimes of Graphidaceae may be the result of the longer evolutionary history. In this family, evolutionary relationships have very little correlation with diversification regimes. For example, the *Myriotrema* clade (regime G3) has a more similar regime to the distantly related Fissurinoideae and Thelotremateae (G1) than the more closely related *Ocellularia* clade (G5). With a longer time span to diversify, it is possible that each lineage encountered a unique set of conditions in different habitats, which led to different diversification dynamics. Unlike groups in Parmeliaceae where groups tend to occur across a wide range of habitats and geographical regions, each major clade in Graphidaceae occurs in its somewhat unique habitat. Species in the *Ocellularia* clade, for example, are more likely to occur in shaded, more protected tropical forest, while taxa in Graphideae typically occur in more exposed areas^23^. Differences in current habitat preferences among clades suggest that these lineages have diverged and may have become differentiated in their habitat types, and the observed speciation rates are the results of habitat-specific processes as opposed to global processes that affect the whole family. It is also possible that the seemingly lower heterogeneity in Parmeliaceae is skewed by exceptionally high rates in *Usnea* (P2) and *Xanthoparmelia* (P3), which can make the other existing regimes (lumped together as P1) in the family appear more homogeneous by comparison. At this point, cohort analysis is still in its early stage^24^ and has a limited explanatory power beyond visualising the patterns of diversification.

We did not observe any clear correlation between speciation rates of Graphidaceae and global temperatures in the past 180 million years. Even though paleoclimate data and our estimates of speciation rates may not be extremely accurate in respect to the absolute geological time, lineages in Graphidaceae have several features that do not necessary respond to climate variability. First, the vast majority of the lineages is tropical^25^. In comparisons to other ecosystems, the tropics have been the most stable habitat through the deep history, and therefore a lineage diversifying in the tropics may not experience as much climatic fluctuation at a global scale^26^. Extratropical lineages in the family, such as *Diploschistes* and *Topeliopsis*, have only recently emerged ( ≤10 MYA)^27^ and did not exhibit any clear pattern in responses to climatic events at this scale. It is also possible that diversification events in Graphidaceae were triggered by other factors beyond climate. For example, diversification of the two most diverse clades (Graphideae and *Ocellularia*) coincide with major historical events. The Graphideae clade, consisting of mostly epiphytic species, began diversifying at 130 MYA, not long after the origin of angiosperms at approximately 140 MYA^28^, while the *Ocellularia* clade diversified at 65 million years ago, which coincide with the Cretaceous-Paleogene (K-Pg) boundary, as well as the expansion of angiosperms-dominated tropical rainforests^29^. It is possible that diversification of Graphidaceae responded more to the diversification of host plants which are mostly angiosperms. Moreover, a unique structure, called “columella”, in the *Ocellularia* clade, was shown to have a positive association with diversification rates in a Binary State Speciation Extinction (BiSSE) analysis and potentially acted as a key innovation for a radiation in the family^30^.

Elevated speciation rates in Parmeliaceae appear to be the results of relatively more recent events, as virtually all higher rates and three major rate shifts occurred in the lineages that are less than 30 million years old (*Usnea*, *Bryoria*, *Hypogymnia*, *Hypotrachyna*, *Xanthoparmelia*, *Flavoparmelia*, and *Parmotrema*). In particular, *Usnea* and *Xanthoparmelia* have nearly 2-3 times higher rates than the family average, which suggests that they play a major role in contributing to the hyperdiversity of the family. In comparisons to all other genera, *Usnea* and *Xanthoparmelia* have developed unique sets of strategies to exploit a much wider range of habitats. Informally known as “old man beard lichens,” *Usnea* species have a bushy or pendulous growth form that allows them to minimally attach to the substrate surface, while exploiting a vast “empty” three-dimensional space to capture extra carbon dioxide and moisture^31^,^32^. These lichens also produce predominantly usnic acid, which was shown to reflect extra sunlight and consequently protect algal cells inside^33^. Abilities to capture more moisture in the empty space and remain protected from sunlight may in part explain their success during the drying period of the Oligocene-Miocene transition (20-30 MYA), which was when cold temperate forests started to evolve and saw an increase in a number of deciduous taxa of angiosperms^34^.

With more than 800 described species^35^, *Xanthoparmelia* is unique in Parmeliaceae for including almost exclusively rock- or soil-inhabiting species in semi-arid environments, although a smaller proportion of species can be found in a wide range of habitats, from tropical to alpine zones^36^,^37^,^38^. The radiation within this group was hypothesised to be associated with the shift to a drier habitat^39^, the switch to rocks and soils as a substrate^40^, and the emergence of novel arid habitats in the Southern Hemisphere from the splitting of modern Australia from South America^41^. Our results are consistent with these hypotheses, in which we observed the correlation between the increased speciation rate in *Xanthoparmelia* and the expansion of drylands during the Oligocene-Miocene transition. While the exact mechanisms of how *Xanthoparmelia* manages to be extremely successful in these habitats are still not clear, a number of their secondary metabolites have been shown to have anti-oxidant properties^42^,^43^ – an important quality for organisms in an environment with high sunlight exposure^44^.

In this study, we have demonstrated that two families of lichenized fungi may share a similar strategy of adopting algal symbiosis, but can become equally hyper-diverse through different evolutionary routes. While lichenization may in general lead to higher diversification rates^17^, we provide additional evidence for the hypothesis that different mechanisms and evolutionary processes may lead to similar levels of species diversity in the two most diverse families of lichenized fungi. We hope that our study generates more interest in using modern analytical methods to study evolution of these understudied, but hugely important groups of organisms.

## Methods

### Chronogram construction

For Graphidaceae, we used the alignment from a previous study on trait evolution within Graphidaceae^25^. Four molecular markers (mtSSU, nuLSU, *RPB-1*, *RPB-2*) from 219 taxa from all of the major clades were assembled^45^. Five taxa from the genus *Gyalecta* were chosen as outgroups. As for Parmeliaceae, we used the six-gene alignment from the most comprehensive dataset available from Parsys2 project, containing six markers (ITS, mtSSU, nuLSU, *RPB-1*, *Tsr1, Mcm7*)^46^ from 312 taxa from all of the major clades^19^,^46^.

For both families, we employed a Bayesian analysis in the program BEAST v 1.6.1^47^ to estimate divergence times with unlinked substitution models (GTR + I) across the loci and a Birth-Death process tree prior. The analysis was run for 100 million generations with parameter values sampled every 5000 generation. The ESS values of the estimated parameters were checked with the program Tracer v 1.3.1 for convergence. Discarding the first 25% of the posterior trees as a burn-in, we then calculated a maximum credible tree using Tree Annotator v 1.6.1. In the case of Graphidaceae, we set the rate of molecular clock for *RPB-1* using the estimate from a study in Lecanoromycetes^41^ and allowed BEAST to estimate the rates for other loci with a uniform prior. For Parmeliaceae, the parameters and setting for the analysis of divergence time followed the another study on the global analysis of the family, which used three fossil calibration points and a constraint at a crown node^41^,^46^. Subsequent analyses and visualisation were performed in the statistical programming language R with associated packages. The resulting maximum credible trees are available on TreeBase (http://purl.org/phylo/treebase/phylows/study/TB2:S16657) and in the supplementary figures 1-2 online.

### Diversification analysis

We used the Bayesian Analysis of Macroevolutionary Mixtures (BAMM) version 2.0 to investigate diversification dynamics in these two large families of lichenized fungi^20^. BAMM is a freely available program developed specifically to detect heterogeneity in rates of diversification processes (speciation and extinction) from a time-calibrated tree. In contrast to other methods, such as MEDUSA (Modeling Evolutionary Diversity Using Step AIC)^48^, which assumed one rate regime across the whole tree, BAMM employs reversible jump Markov chain Monte Carlo to explore all possible distinct rate-shift configurations on the tree and return a posterior space of all these configurations for further inference. The algorithm and subsequent analyses have been described in details elsewhere^4^,^20^,^24^, as well as the online documentation at http://bamm-project.org.

For each of the two studied family, we used the newly constructed chronogram as an input for the BAMM analysis. In order to account for non-random incomplete sampling, we calculated the proportion of species sampled at the clade level, as recognised in previous phylogenetic studies^18^,^19^,^25^,^45^,^49^. These proportions were used as inputs for the “SamplesProbsFilename” argument in the control file (supplementary tables 1-2 and supplementary figures 1-2 online). The priors for diversification analyses were set for each tree, using the setBAMMPriors command in the BAMMtools package in R. We ran four parallel Markov chains for 40,000,000 generations and sampled the results every 1000^th^ tree. The output of the programs and subsequent analyses were conducted in R [R Development Core Team 2014], using the BAMMtools package v. 2.0^20^.

After discarding the first 20% of the results, we checked the ESS values for likelihood for convergence with the R-package “coda” [Plummer *et al.* 2005]. From the rest of the output, we then drew “phylorate” plots to depict the speciation rates across the tree, colour-coded by relative values on the trees. The four best rate shift configurations were recovered from a credible shift set with the Bayes Factor criterion of 3 or higher.

In order to visualise the number of different regimes of diversification within the family, we conducted a “cohort analysis”^24^, which calculates a pairwise similarity in diversification rates between any two tips on the tree and plots a heat map of these differences across the tree. A higher number of different colours on this heat map indicated a higher heterogeneity in diversification regimes in the group. Based on these pairwise similarities, we arbitrarily grouped clades together into 4-5 regimes as indicated on the right side of the heat map (Fig. 2).

We also plotted the speciation rate through time to visualise the change of the diversification process at the family level, as well as of the clades where high rates of speciation rates were detected (Graphideae and *Ocellularia* clades in Graphidaceae, and *Xanthoparmelia* and *Usnea* clades in Parmeliaceae). On the same time scale (180-0 million years before present), global temperatures from ice core CO_2_^50^ and δ^18^O isotopes^51^ estimates were also calculated to as anomalies from the mean temperature in 1960-1990 (Fig 3).

## Author Contributions

E.K. and H.T.L. designed the research and wrote the paper. R.L, P.K.D and A.C. assembled molecular data and conducted phylogenetic analyses. E.K. performed the diversification analysis. S.L. and M.N. assisted in the diversification analysis and revision of the manuscript.

## Additional Information

**How to cite this article**: Kraichak, E. *et al.* A Tale of Two Hyper-diversities: Diversification dynamics of the two largest families of lichenized fungi. *Sci. Rep.* **5**, 10028; doi: 10.1038/srep10028 (2015).

## Supplementary Material

Supplementary Information
