# Supplementary material for: A Tale of Two Hyper-diversities: Diversification dynamics of the two largest families of lichenized fungi
Source: Sci Rep. 2015 May 6;5:10028. doi: 10.1038/srep10028 (PMC4421861; doi:10.1038/srep10028)
Supplement: Supplementary Information [file srep10028-s1.pdf]

Supplementary Materials

A Tale of Two Hyper-diversities: Diversification dynamics of the two largest families of lichenized fungi

Ekaphan Kraichak[1,2], Pradeep K. Divakar[3], Ana Crespo[3], Steven D. Leavitt[1], Matthew P. Nelsen[1,4] ,Robert Lücking[1], H. Thorsten Lumbsch[1]

[1] Science and Education, The Field Museum, 1400 S Lake Shore Drive, Chicago, IL 60605 USA

[2] Department of Botany, Faculty of Science, Kasetsart University, Bangkok, 10900 Thailand

[3] Departamento de Biologia Vegetal II, Facultad de Farmacia, Universidad Complutense de Madrid, Madrid 28040, Spain

[4] Department of Geological and Environmental Sciences, Stanford University, Stanford, CA 94305

Supplementary Figure 1: Maximum Credibility Clade Tree of lichen-forming fungi family Graphidaceae. See node names in supplementary table 1.

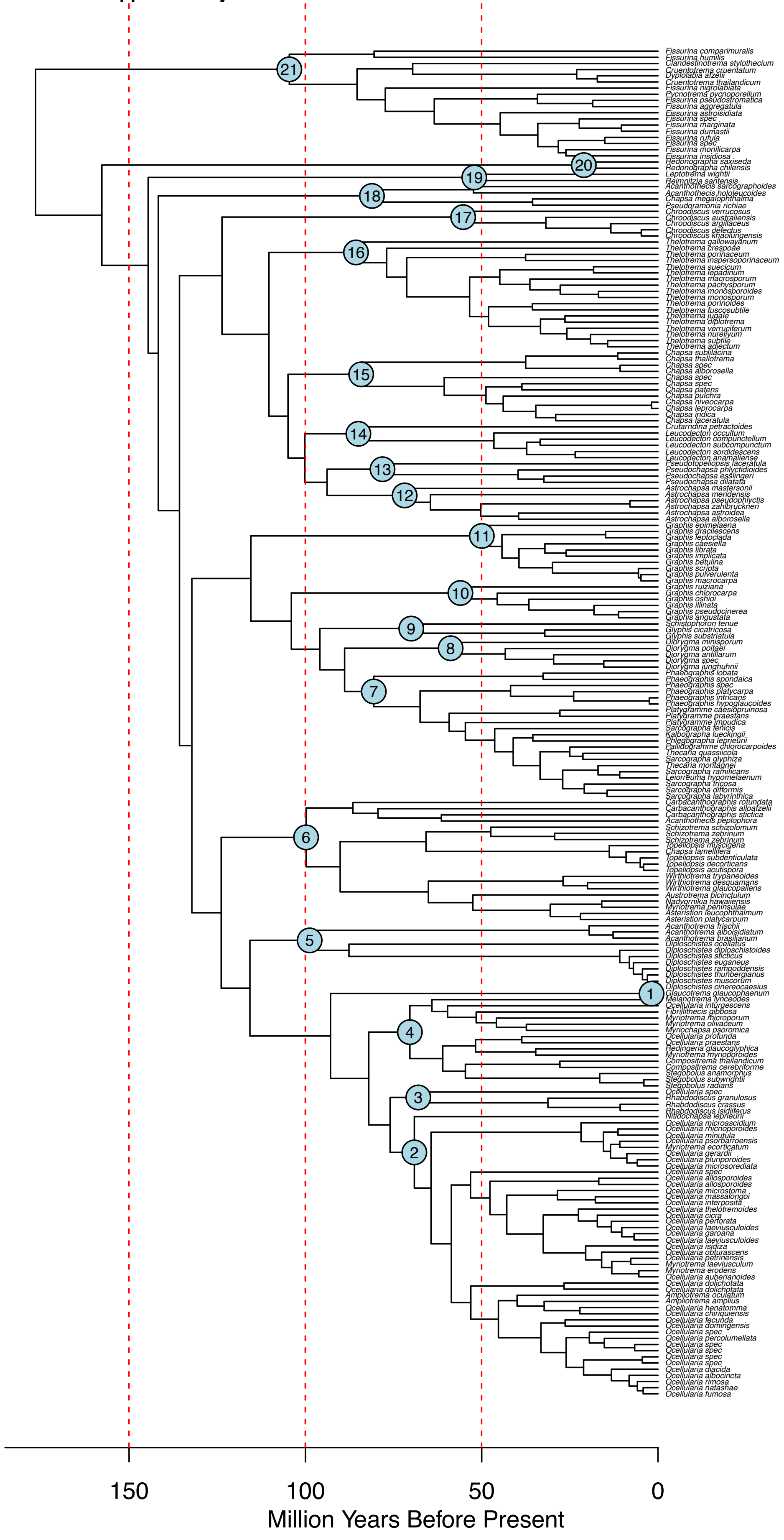

Supplementary Figure 2: Maximum Credibility Clade Tree of lichen-forming fungi family Parmeliaceae. See node names in supplementary table 2.

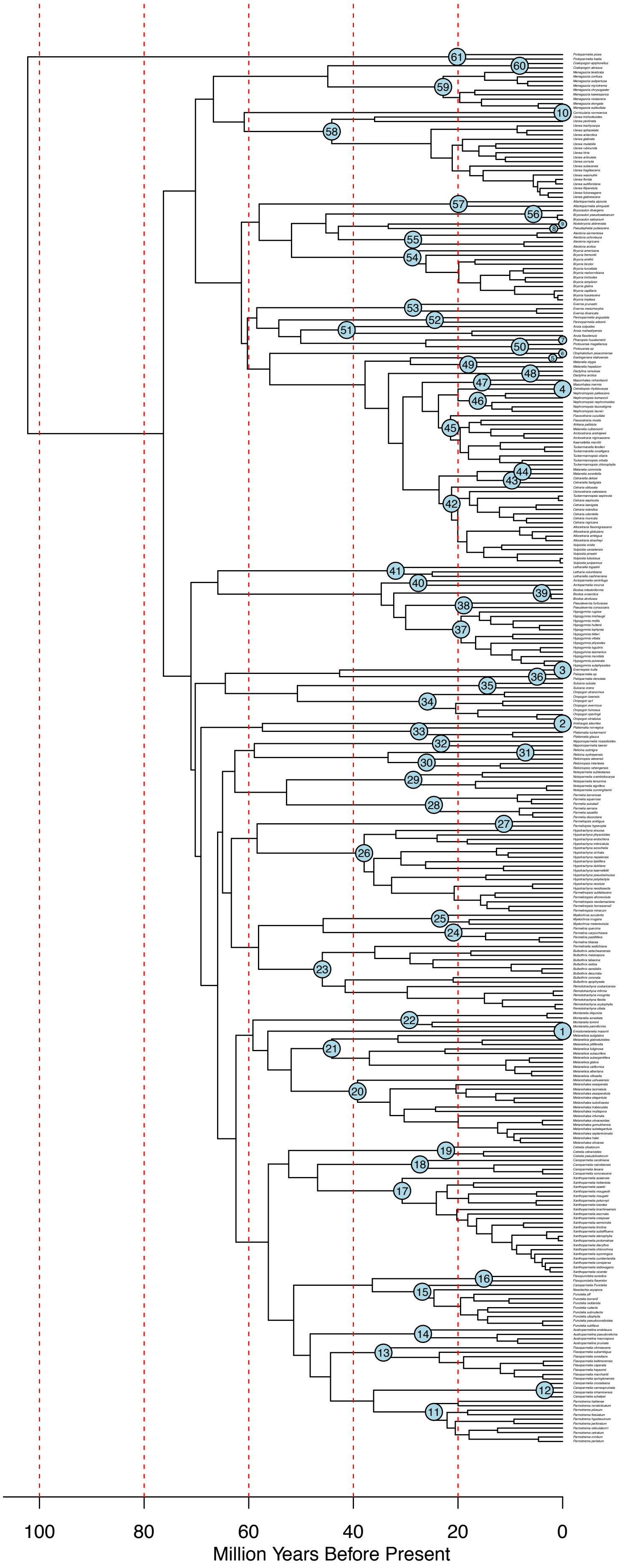

Supplmentary Table 1: Clades and their species richness in Graphidaceae

clade	Node Number	Total Number of Taxa	Number of sampled taxa
Glaucotrema	1	6	1
Ocellularia	2	317	46
Rhabdodiscus	3	43	4
Myriotrema	4	126	15
Diploschisteae	5	39	11
Wirthiotremateae	6	83	20
Phaeographis	7	300	21
Diorygma	8	90	5
Glyphis	9	13	3
Allographa	10	237	6
Graphis	11	314	10
Astrochapsa	12	22	6
Pseudochapsa	13	22	4
Leucodecton	14	28	6
Chapsa	15	58	12
Thelotrema	16	107	18
Chroodiscus	17	14	5
Acanthothecieae	18	63	4
Leptotremateae	19	5	2
Redonographoideae	20	7	2
Fissurinoideae	21	185	18

Supplmentary Table 2: Clades and their species richness in Parmeliaceae

clade	Node Number	Total Number of Taxa	Number of sampled taxa
Emodomelanelia	1	1	1
Imshaugia	2	7	1
Everniopsis	3	1	1
Cetreliopsis	4	7	1
Esslingeriana	5	1	1
Omphalodium	6	4	1
Phacopsis	7	35	1
Pseudophebe	8	2	1
Nodobryoria	9	3	1
Conicularia	10	1	1
Parmotrema	11	300	10
Canoparmelia1	12	20	4
Flavoparmelia	13	32	8
Austroparmelina	14	13	4
Punctelia	15	49	10
Flavopunctelia	16	5	2
Xanthoparmelia	17	820	22
Canoparmelia2	18	20	4
Cetrelia	19	15	3
Melanohalea	20	22	15
Melanelixia	21	15	10
Montanelia	22	9	4
Bulbothrix	23	73	15
Parmelina	24	9	4
Myelochroa	25	30	3
Hypotrachyna	26	213	19
Parmeliopsis	27	3	2
Parmelia	28	41	6
Notoparmelia	29	16	5
Relicinopsis	30	5	3
Relicina	31	54	2
Nipponoparmelia	32	4	2
Platismatia	33	11	3
Oropogon	34	40	7
Sulcaria	35	4	2
Psiloparmelia	36	13	2
Hypogymnia	37	90	13
Pseudoevernia	38	4	2
Brodoa	39	3	3
Arctoparmelia	40	5	2
Letheria	41	17	3
Cetraria	42	32	18
Cetrariella	43	4	2
Melanelia2	44	6	2
Tuckermaniopsis	45	38	12
Nephromopsis	46	21	5

clade	Node Number	Total Number of Taxa	Number of sampled taxa
Masonhalea	47	2	2
Dactylina	48	2	2
Melanelia1	49	7	2
Protousnea	50	8	2
Anzia	51	34	3
Pannoparmelia	52	5	2
Evernia	53	10	3
Bryoria	54	51	12
Alectoria	55	9	4
Bryocaulon	56	4	3
Allantoparmelia	57	3	2
Usnea	58	350	19
Menegazzia	59	70	9
Coelopogon	60	2	2
Protoparmelia	61	15	2
